# Genetic identification of tissues and cell types underlying attention-deficit/hyperactivity disorder

**DOI:** 10.3389/fpsyt.2022.999007

**Published:** 2022-08-26

**Authors:** Wen-Qiong Wei, Hong Sun, Ya-Juan Chen, Xiao-Wen Liu, Rui Zhou, Yi Li, Xin-Wen Liu

**Affiliations:** ^1^Department of Digestive, Wuhan Children's Hospital (Wuhan Maternal and Child Healthcare Hospital), Tongji Medical College, Huazhong University of Science and Technology, Wuhan, China; ^2^Medical Laboratory, Wuhan Children's Hospital (Wuhan Maternal and Child Healthcare Hospital), Tongji Medical College, Huazhong University of Science and Technology, Wuhan, China; ^3^Department of Breast Surgery, Wuhan Children's Hospital (Wuhan Maternal and Child Healthcare Hospital), Tongji Medical College, Huazhong University of Science and Technology, Wuhan, China; ^4^Department of General Surgery, Wuhan Children's Hospital (Wuhan Maternal and Child Healthcare Hospital), Tongji Medical College, Huazhong University of Science and Technology, Wuhan, China; ^5^Department of Orthopaedics, Wuhan Children's Hospital (Wuhan Maternal and Child Healthcare Hospital), Tongji Medical College, Huazhong University of Science and Technology, Wuhan, China; ^6^Department of Psychiatry, Wuhan Mental Health Center, Wuhan, China; ^7^Nursing Department, Wuhan Children's Hospital (Wuhan Maternal and Child Healthcare Hospital), Tongji Medical College, Huazhong University of Science and Technology, Wuhan, China

**Keywords:** ADHD, GWAS, gene expression, tissues enrichment, cell type enrichment

## Abstract

**Background:**

Genome-wide association studies (GWASs) have identified numerous genetic variants associated with attention-deficit/hyperactivity disorder (ADHD), which is considered highly genetically heritable. However, because most of the variants located in the non-coding region of the human genome, the onset of ADHD requires further exploration.

**Methods:**

The risk genes involved in ADHD were identified by integrating GWAS summary data and expression quantitative trait locus (eQTL) data using summary-data-based Mendelian randomization (SMR) method. We then used a stratified linkage disequilibrium score regression (LDSR) method to estimate the contribution of ADHD-relevant tissues to its heritability to screen out disease-relevant tissues. To determine the ADHD-relevant cell types, we used an R package for expression-weighted cell type enrichment (EWCE) analysis.

**Results:**

By integrating the brain eQTL data and ADHD GWAS data using SMR, we identified 247 genes associated with ADHD. The LDSR applied to specifically expressed genes results showed that the ADHD risk genes were mainly enriched in brain tissue, especially in the mesencephalon, visual cortex, and frontal lobe regions. Further cell-type-specific analysis suggested that ADHD risk genes were highly expressed in excitatory neurons.

**Conclusion:**

The study showed that the etiology of ADHD is associated with excitatory neurons in the midbrain, visual cortex, and frontal lobe regions.

## Introduction

Attention-deficit/hyperactivity disorder (ADHD) is a commonly diagnosed neurodevelopmental disorder which is usually characterized by inattention, impulsivity, and hyperactivity. It has been reported that approximately 5% of children and 2.5% of adults in worldwide population were affected by ADHD (1). Moreover, a large proportion of ADHD patients also have other comorbid psychiatric disorders, such as anxiety, emotional, and educational disorders. Research has shown that ADHD patients have a high risk of educational and occupational failure, accidents, criminality, social disability, divorce, suicide, and premature death (2).

Currently, ADHD is diagnosed in clinics using the DSM-5 or ICD10. Furthermore, clinical therapeutics for ADHD usually require long-term care, and the recurrence of symptoms is common (1). Therefore, determining the etiology of ADHD is crucial to improving clinical diagnoses and treatments for ADHD.

Accumulating epidemiological and clinical evidence has shown that ADHD is affected by both genetic and environmental factors. Genetic studies of identical twins have reported that the heritability of ADHD is approximately 0.76 (3–5), which indicates that genetic factors are primarily responsible for the onset of ADHD. However, ADHD has also been demonstrated to undergo polygenic transmission and be a complex neurodevelopmental disorder that can be affected by both common and rare variants (2). Numerous genome-wide association studies (GWAS) have identified multiple single nucleotide polymorphisms (SNPs) related to ADHD susceptibility (6, 7). Intriguingly, most of these genetic loci are positioned in the non-coding region of the human genome. Furthermore, because SNPs likely influence complex traits in a tissue- and cell-type-specific manner, further explorations of the relevant biological mechanisms of SNPs may become even more complicated (8).

Extensive research has shown that trait-associated SNPs in non-coding regions regulate the expression of genes to further their involvement in ADHD etiology (9–11). Based on these studies, we applied summary-data-based Mendelian randomization (SMR) to the analysis of integrating GWAS and expression quantitative trait locus (eQTL) data to identify the risk genes of ADHD. Additionally, we used the stratified linkage disequilibrium score regression (LDSR) method (12), also known as LDSC, to estimate the contribution of various tissues to the heritability of ADHD and identify ADHD-relevant tissues. Finally, an R package for expression-weighted cell type enrichment (EWCE) analysis (13) was used to perform cell-type enrichment analysis to identify ADHD-relevant cell types.

## Materials and methods

### Attention-deficit/hyperactivity disorder genome-wide association study data

Attention-deficit/hyperactivity disorder genome-wide association study data were obtained from the meta-analysis by Demontis et al. (6), which included 20,183 ADHD cases and 35,191 controls from 12 cohorts. The samples were mainly from European populations. After quality control, 8,047,421 SNPs were remained for final meta-analyze. In total, 304 genetic variants in 12 genomic loci were shown to be significantly related to ADHD (*P* < 5 × 10^−8^). The GWAS summary statistics data are available for download from the Psychiatric Genomics Consortium website (https://www.med.unc.edu/pgc/results-and-downloads).

### Summary-data-based Mendelian randomization analysis

We applied SMR to integrate ADHD GWAS data and eQTL data to identify the ADHD risk genes. In the SMR analysis, ADHD-relevant SNPs were used as instrumental variables (IVs) to test the causal effect of the exposure (gene expression) on the outcome (ADHD) (14). Detailed information about the theoretical principle, model hypothesis, and algorithm implementation of SMR is available in the original publication (14). BrainMeta v2 cis-eQTL summary data were obtained from the SMR website (https://yanglab.westlake.edu.cn/software/smr/#DataResource), which contained cis-eQTL information using RNA-seq expression and genotype data of 2,865 brain cortex samples from 2,443 unrelated European individuals. Qi et al. (15) meta-analyzed the brain frontal cortex data from GTEx (16), CMC (17), and ROSMAP (18). This dataset only contained *cis*-eQTL summary statistics for 16,704 genes and about 11.6 million SNPs with minor allele frequency larger than 0.01. We also downloaded the eQTL data (16) from 13 GTEx (v7) brain tissues for different brain region SMR analysis. The GTEx project aims to characterize variation on gene expression levels across different human tissues. The v7 release of GTEx contains a total of 11,688 samples, mainly European ancestry, from 53 tissues of 714 donors within an age range from 20 to 79 years. For SMR analysis, we used the default parameters, and to decrease the false positive rate, *P*_SMR_ < 0.01 was set as the significant level. To further test the heterogeneity of the IVs, we performed heterogeneity in dependent instruments (HEIDI) to filter out results with *P*_HEIDI_ < 0.01.

### Synapse gene ontology annotations

All risk genes were uploaded to the Synapse Gene Ontology (SynGO) website (https://syngoportal.org/) (19) for further annotation. The SynGO cellular component terms are displayed in the sunburst plot.

### LDSC applied to specifically expressed genes

The LDSC applied to specifically expressed genes (LDSC-SEG) method was used to identify disease-relevant tissues using gene expression data from different tissues and GWAS summary statistics (12). For each gene, the *t*-statistics was first calculated for the specific expression in the target tissue during the LDSC-SEG analysis. The genes were then arranged in descending order according to the *t*-statistics, and those within the top 10% were defined as the tissue-specific gene set. As an additional step, a 100 kb window was added to both sides of the transcription region of the genes in the tissue-specific gene set to construct tissue-specific genome annotations. The LDSC-SEG analysis was then applied to the different annotated groups to estimate the contribution of various tissues or cells to the target phenotypic heritability. Detailed information about the theoretical principle, model hypothesis, and algorithm implementation of the LDSC-SEG analysis is provided in the original publication (12). We downloaded the microarray-based gene expression data of various tissues and cell types from Franke's lab, which contained 152 tissues or cell types originating from 37,427 human samples for about 3,404 genes (https://alkesgroup.broadinstitute.org/LDSCORE/LDSC_SEG_ldscores) (20, 21). All datasets were processed using ldsc.py (version 1.0.1) with default parameters. For the outcomes, *P*_LDSC−SEG_ < 0.05 was considered significant.

### Expression-weighted cell type enrichment analysis

Single-cell transcriptome expression data are used to generate a probability distribution genes list containing the average expression in a cell of interest. The gene expression level is then estimated using the target gene set to check whether it is higher than that of randomly chosen genes in the cell of interest (13). We used data of single-cell gene expressions of human neocortical development during mid-gestation from Polioudakis et al. and Trevino et al. (22, 23). Polioudakis et al. obtained approximately 40,000 cortical single-cell expression data from four donors at mid-gestation week 17–18 using Drop-seq. Using dimension reduction analysis and cell-type mapping, they screened out 16 transcriptionally distinct cell types, including excitatory neurons, inhibitory neurons, interneurons, and microglia (22). Trevino et al. created a gene expression atlas using 10× genomics from four primary samples at post-conception weeks 16, 20, 21, and 24. Overall, they obtained 57,868 single-cell transcriptomes and divided these cells into 23 clusters (23). Using these two datasets, combined with the risk gene sets retrieved from the SMR analysis, we were able to identify ADHD associated tissues using risk genes from SMR analysis. Details about the EWCE principle are provided in the original publication (13). The data analysis procedure is also described at https://github.com/NathanSkene/EWCE/. *P*_EWCE_ < 0.05 was considered as significant.

## Results

### Summary-data-based Mendelian randomization integrative analysis

**Figure 1:**
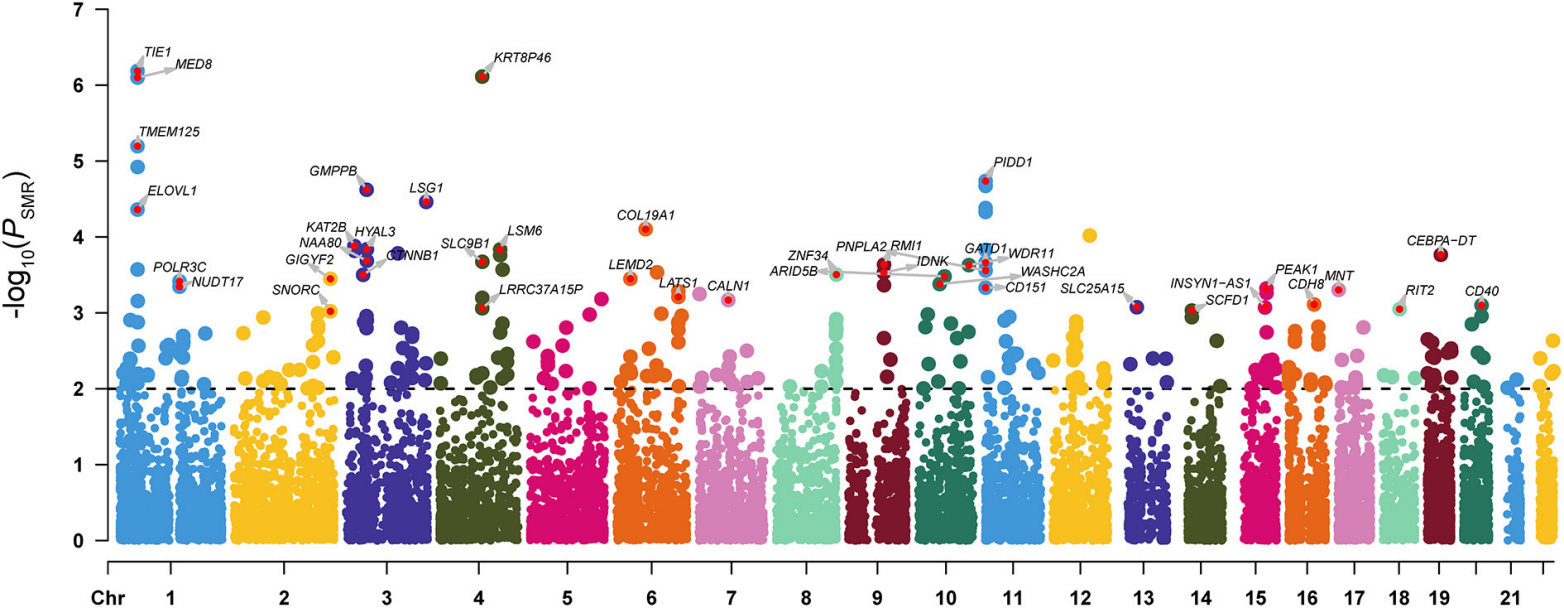
Manhattan plot of the SMR analysis integrating ADHD GWAS data with the BrainMeta eQTL data. *P*-value indicates the statistical significance of the SMR analysis results. The dotted line represented the SMR significant threshold (*P*_SMR_ < 0.01). For genes with *P*_SMR_ < 0.001 and *P*_HEIDI_ > 0.01, we also marked the gene names in the figure.

**Figure 2:**
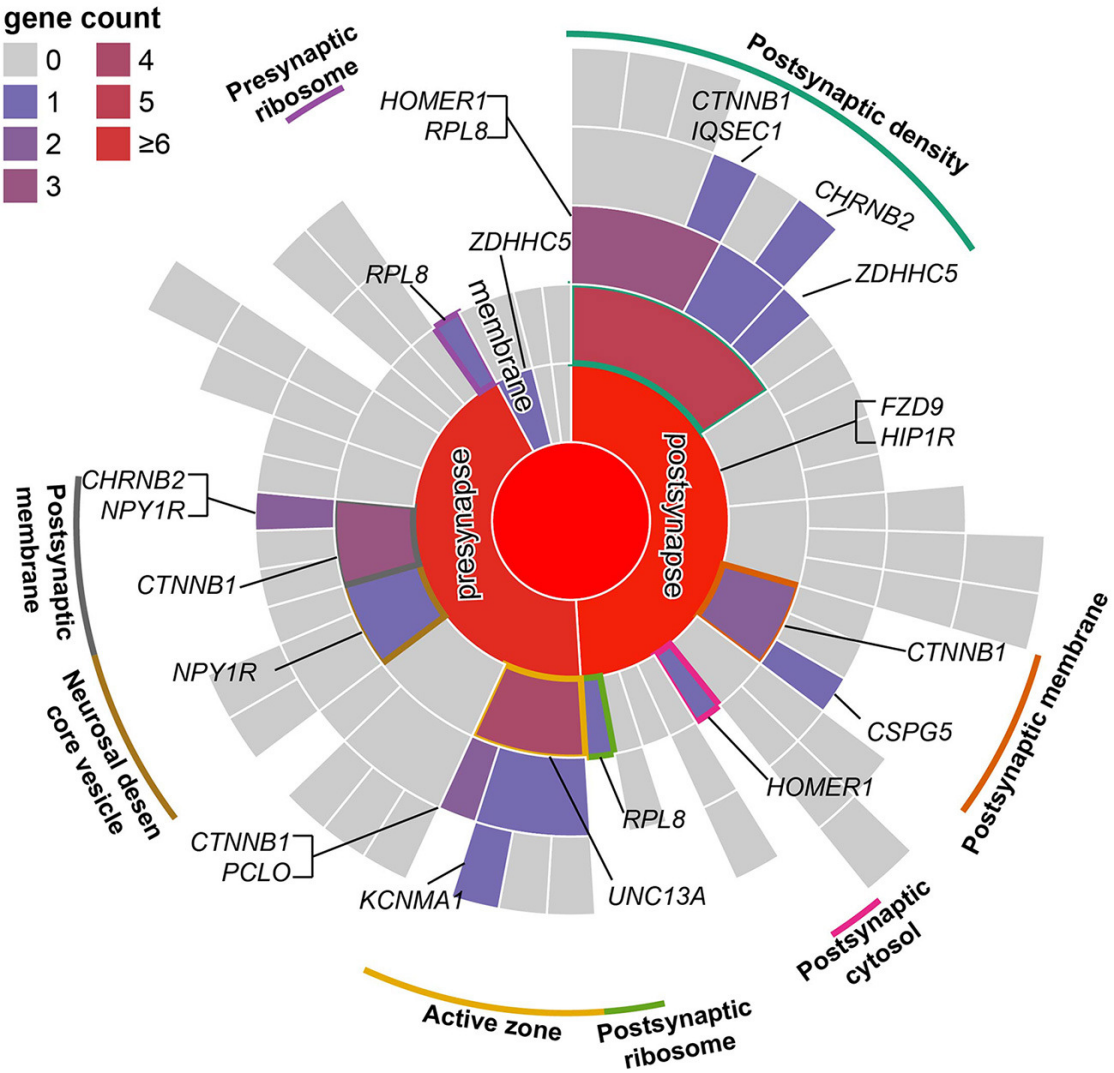
Mapping of SMR risk genes to synaptic locations using SynGO. According to the location of genes in the synapse, genes were divided into presynaptic, postsynaptic, synaptic cleft, synaptic membrane, and extrasynaptic genes. Similar to a directed acyclic graph of GO, terms in the outer ring are a subset of those in the adjacent inner ring.

The SMR analysis identified 247 and 129 risk genes for ADHD using BrainMeta v2 eQTL data and 13 GTEx brain tissues data, respectively (Figure 1 and Supplementary Tables S1, S2). In total, 336 genes were identified to be associated with ADHD. Intriguingly, multiple risk genes were related to neurodevelopment. These genes included *CAMK1D*, a member of the calcium/calmodulin-dependent protein kinase 1 family that is responsible for the growth of hippocampal neurons dendrites; *KCTD16*, a subunit of the gamma-aminobutyric acid-B receptor, which is involved in the excitability of dendrites; and *CTNNB1* alongside the transcription factor LEF1/TCF, which promote the expression of genes responsible for developmental processes, such as neurogenesis and synaptic plasticity (24). Moreover, the result of the SynGO annotations showed that 16 genes were involved in the formation of synapses (Figure 2).

### LDSC applied to specifically expressed genes analysis to identify ADHD-relevant tissues

**Figure 3:**
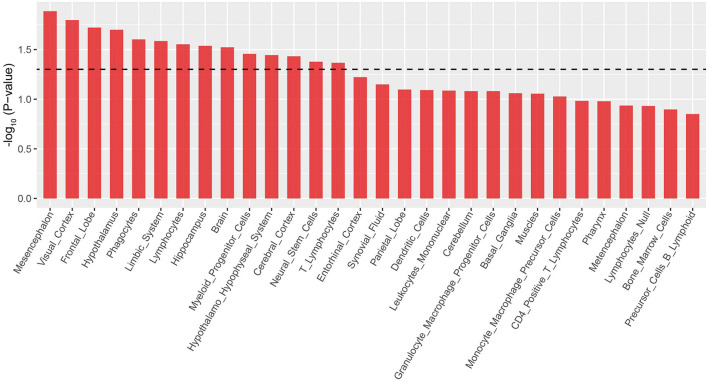
LDSC-SEG analysis results of ADHD-relevant tissues. Only the top 30 tissues are shown according to the descending order of log_10_ (*p*-value).

The brain is a complex organ that comprises numerous brain regions which perform different functions. Therefore, it is important to analyze disease-associated brain regions. Results of the enrichment analysis using GWAS data in multiple tissues showed that the ADHD risk loci were significantly enriched in the mesencephalon (*P* = 0.01), visual cortex (*P* = 0.02), and frontal lobe (*P* = 0.02) (Figure 3 and Supplementary Table S3). This result suggested that risk genes are involved in the onset of ADHD *via* the regulation of gene expression in these brain regions. Moreover, our results further confirmed that ADHD is a brain-associated neurodevelopmental disorder.

### Expression-weighted cell type enrichment analysis to identify ADHD-relevant cell types

**Figure 4:**
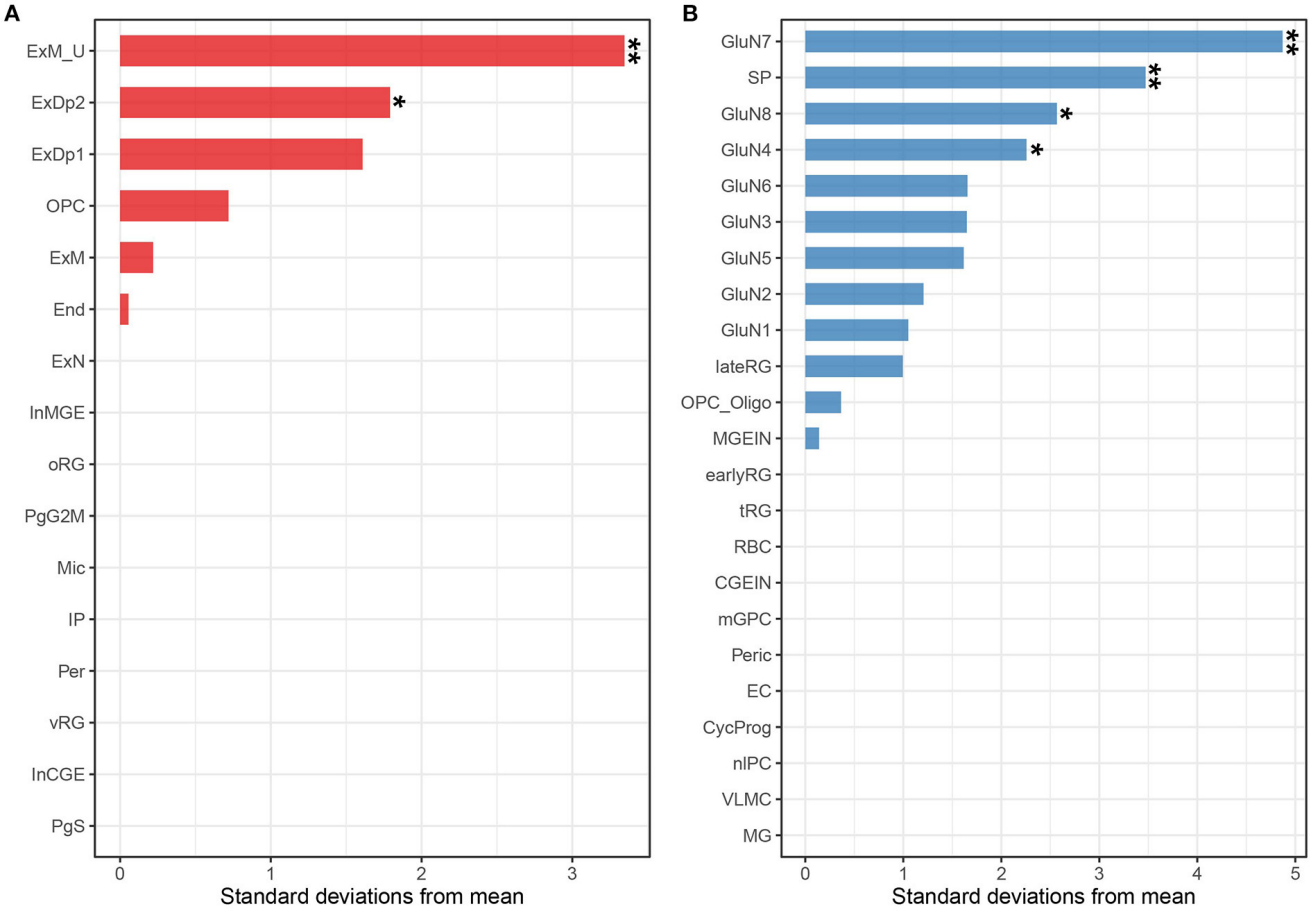
Associations between ADHD and cell types from the human fetal neocortex using scRNA-seq data from Polioudakis et al. (22) **(A)** and Trevino et al. (23) **(B)**. Tissues labeled with two asterisks indicate Bonferroni adjusted significance (*P* < 0.05 for the Polioudakis et al. (22) data; *P* < 0.05 for the Trevino et al. (24) data). Tissues labeled with one asterisk indicate the nominal significance (*P* < 0.05). End, endothelial cell; ExDp1, excitatory deep layer 1; ExDp2, excitatory deep layer 2; ExM, maturing excitatory; ExM-U, maturing excitatory upper enriched; ExN, migrating excitatory; InCGE, interneuron; InMGE, interneuron MGE; IP, intermediate progenitor; Mic, microglia; OPC, oligodendrocyte precursors; oRG, outer radial glia; Per, pericyte; PgG2M, cycling progenitors (G2/M phase); PgS, cycling progenitors; vRG, ventricular radial glia; GluN, glutamatergic neuron; SP, subplate; nIPC, neuronal intermediate progenitor cell; lateRG, late radial glia; earlyRG, early radial glia; VLMC, leptomeningeal cells; tRG, truncated radial glia; RBC, red blood cells; Peric, pericytes; OPC_oligo, OPC and oligodendrocyte; mGPC, multipotent glial progenitor; CycProg, cycling progenitors.

Although we found that the risk genes were highly expressed in brain-associated tissues, the brain contains thousands of different cell types with distinct gene expression patterns (25), and similar to the tissue specificity of gene expression, cell specificity should not be ignored. We considered that risk genes may only exert function in certain cell types (26, 27). Therefore, we applied EWCE using the R package to perform cell-type-specific analysis. As mentioned earlier, risk genes may be involved in neurodevelopment because ADHD is a neurodevelopmental disorder. Thus, we selected single-cell gene expression data from the neocortex at mid-gestation (i.e., gestation weeks 17–18). Expression-weighted cell type enrichment analysis performed on the risk gene set showed that the ADHD risk genes were highly expressed in mature excitatory neurons in the upper layer (ExM-U, *P*_EWCE_ = 1.6 × 10^−3^) and excitatory neurons in deep layer 2 (ExDp2, *P*_EWCE_ = 0.04) (Figure 4A and Supplementary Table S4) (22). The cell-type-specific analysis using another single-cell dataset also highlighted excitatory glutamatergic neurons in ADHD (Figure 4B and Supplementary Table S5) (23). Taken together, the results indicated that risk genes are involved in the onset of ADHD by affecting the relevant gene expressions of the excitatory cortical neurons.

## Discussion

Attention-deficit/hyperactivity disorder is a polygenic and complex heritable disorder, whose heritability is consisted of many common variants with small effects and relatively small number of rare variants with large effects. Most common variants identified by GWAS were located in non-coding regions (2, 6), with no influence on the protein sequence. We hypothesized that these common variants play a role in the onset of ADHD by regulating the expression of ADHD-relevant genes.

In this study, we collected ADHD GWAS data, brain eQTL data, tissue expression profile, and cortical single-cell sequencing data to identify the ADHD-associated risk genes, relevant tissues, and cell types. We screened out 336 ADHD-associated risk genes, including multiple neurodevelopment-related genes and some of these genes, like KIZ and CTNNB1, were also indicated to be related to ADHD in previous studies (28, 29). The tissue-specific analysis showed that the risk genes were highly expressed in brain tissues, especially in the mesencephalon, visual cortex, and frontal lobe regions. Moreover, the cell-specific analysis showed that ADHD risk genes are highly expressed in excitatory neurons.

The LDSC-SEG results for the integrated tissue expression profile and ADHD GWAS data revealed that multiple brain regions were involved in the onset ADHD. Although the results were not adjusted by multiple correction, the most significant tissues were located in the brain. Therefore, we consider our results reliable. However, because of the high similarity in expression profiles among various brain tissues, we were unable to identify specific ADHD-associated brain tissues using the current methods.

Although hundreds of risk genes for ADHD have been identified using SMR analysis, identifying the specific brain regions in which these risk genes exert its function requires further exploration because of cellular heterogeneity. Previous studies have shown that, in contrast to neurological disorders, GWAS data of psychiatric disorders map primarily onto neurons, rather than glial cells (26, 30). This is in line with our findings that ADHD risk genes are highly expressed in excitatory cortical neurons, which indicated that these genes affect the biological function of excitatory neurons and, further contribute to etiology of ADHD.

Furthermore, several researchers consider ADHD as an omnigenic disorder, whereby all genes contribute to the onset of ADHD (2, 31). The pathogenic genes can be divided into two categories: a small proportion of directly ADHD-related genes, referred to as “core genes” and a large proportion of “peripheral genes,” which function *via* the regulation of the “core genes.” According to this notion, the majority of genes identified in our study likely belong to the “core genes.” In addition, we screened out multiple ADHD-associated risk genes that were also involved in neurodevelopment, which demonstrated that ADHD is a neurodevelopmental disorder (1, 32, 33). Furthermore, the risk SNPs of ADHD may cause neurodevelopmental disorders by regulating the expression of genes related to neurodevelopment; thus, these SNPs are also likely involved in the etiology of ADHD.

## Limitations

We summarized several limitations. First, GWAS risk SNPs can exert its function not only through regulation of gene expression but also through other pathways (34), such as splicing (35), histone modification (36), and chromatin accessibility (37). Second, we did not perform multiple-test correction for the SMR analysis, which may have increased the potential of false positives. However, given that the current GWAS only explained about one-third of the heritability for ADHD, the number of risk loci identified by current GWAS were far from saturation (2). Therefore, instead of multiple-test correction, we filtered the false-positive results using a *P*_SMR_ threshold of 0.01. Finally, the ADHD-associated cell types identified in this study require further validation in other brain regions. As additional single-cell expression maps of brain regions become available, we plan to continue to study ADHD-associated cell types in other brain regions.

## Data availability statement

The datasets presented in this study can be found in online repositories. The names of the repository/repositories and accession number(s) can be found in the article/Supplementary material.

## Author contributions

W-QW and HS designed the study and drafted the manuscript. Y-JC, X-WL, and RZ analyzed the data. YL and X-WL revised the manuscript and provided expert consultation on the design of the study. All authors critically reviewed the manuscript and approved of it in its final form.

## Funding

This research was supported by Wuhan Municipal Health Research Fund (WG17M01 to YL).

## Conflict of interest

The authors declare that the research was conducted in the absence of any commercial or financial relationships that could be construed as a potential conflict of interest.

## Publisher's note

All claims expressed in this article are solely those of the authors and do not necessarily represent those of their affiliated organizations, or those of the publisher, the editors and the reviewers. Any product that may be evaluated in this article, or claim that may be made by its manufacturer, is not guaranteed or endorsed by the publisher.
